# Magnetic resonance lung function – a breakthrough for lung imaging and functional assessment? A phantom study and clinical trial

**DOI:** 10.1186/1465-9921-7-106

**Published:** 2006-08-06

**Authors:** Maren Zapke, Hans-Georg Topf, Martin Zenker, Rainer Kuth, Michael Deimling, Peter Kreisler, Manfred Rauh, Christophe Chefd'hotel, Bernhard Geiger, Thomas Rupprecht

**Affiliations:** 1 University Children's Hospital, University Erlangen-Nuremberg, Loschgestr. 15, 91054 Erlangen, Germany; 2 Siemens medical solutions, Henkestr. 127; 91052 Erlangen, Germany; 3 Siemens Corporate Research; 755 College Road East, Princeton, NJ 08540-6632, USA; 4 Children's Hospital; Preuschwitzer Straße 101, D-95445 Bayreuth, Germany

## Abstract

**Background:**

Chronic lung diseases are a major issue in public health. A serial pulmonary assessment using imaging techniques free of ionizing radiation and which provides early information on local function impairment would therefore be a considerably important development. Magnetic resonance imaging (MRI) is a powerful tool for the static and dynamic imaging of many organs. Its application in lung imaging however, has been limited due to the low water content of the lung and the artefacts evident at air-tissue interfaces. Many attempts have been made to visualize local ventilation using the inhalation of hyperpolarized gases or gadolinium aerosol responding to MRI. None of these methods are applicable for broad clinical use as they require specific equipment.

**Methods:**

We have shown previously that low-field MRI can be used for static imaging of the lung. Here we show that mathematical processing of data derived from serial MRI scans during the respiratory cycle produces good quality images of local ventilation without any contrast agent. A phantom study and investigations in 85 patients were performed.

**Results:**

The phantom study proved our theoretical considerations. In 99 patient investigations good correlation (r = 0.8; p ≤ 0.001) was seen for pulmonary function tests and MR ventilation measurements. Small ventilation defects were visualized.

**Conclusion:**

With this method, ventilation defects can be diagnosed long before any imaging or pulmonary function test will indicate disease. This surprisingly simple approach could easily be incorporated in clinical routine and may be a breakthrough for lung imaging and functional assessment.

## Background

Magnetic resonance imaging (MRI) relies on signals emitted by hydrogen nuclei brought into a magnetic field and stimulated by an electromagnetic radio wave. The usual MRI rule is, the stronger the magnetic field, the stronger the signal. When signal strength is increased imaging is faster, the slices are thinner and the images have a better resolution. With respect to imaging the lung, we have advocated a change of paradigms and could demonstrate why less is more in lung MRI. A low magnetic field strength (0.2 T) yields a lower MR signal but also less artefacts [1-4] This reduction of the artefacts enables us to image lung tissue at low field strength.

Since the lung contains mainly air there is little tissue to give an MR signal, therefore the slice thickness of the images needs to be increased (20 to 200 mm), to produce a clear image [5-8]. We have previously shown that lung MRI is possible and that this technique can be used efficiently for fast and reliable static imaging of the lung [6-8].

Starting point of our approach to a functional (dynamic) assessment of the lung was the observation that in 'real-time' videos of low-field MRI scans grey values of the lung parenchyma changed noticeably during respiration, presumably reflecting the changing tissue/air ratio. This suggested that local ventilation could be measured without a contrast agent and without specific scanner technology, solely on the basis of the data produced by serial low-field MR images.

## Methods

### Theoretical considerations

**Figure 1:**
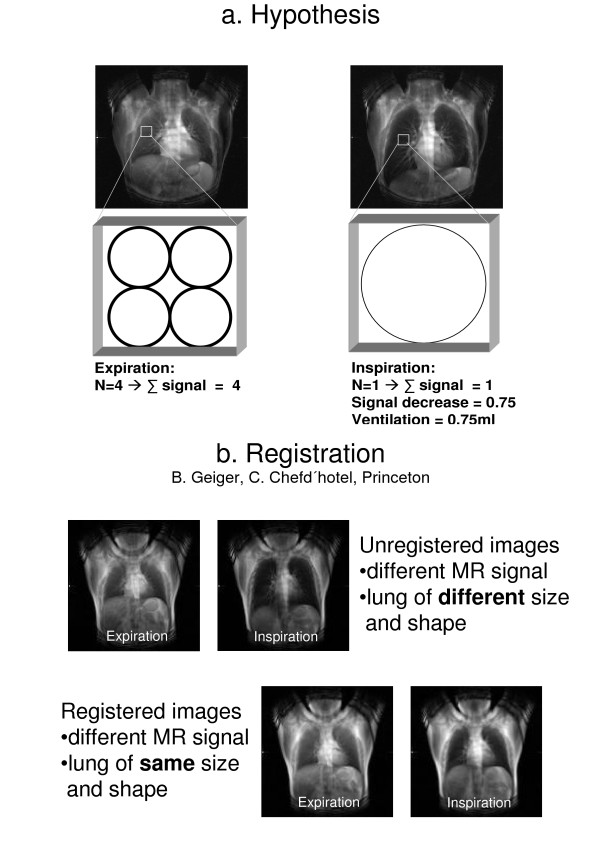
**a**. Native ventilation MR image of one patient during expiration and inspiration. For precise measurement the region of interest would have to move during the respiratory cycle, we therefore include a schematic of our theoretical considerations. The four circles schematically represent four alveoli in a volume (voxel). During inspiration, tissue will be replaced by air causing a lower MR signal as shown on the right. In the same volume now only one of the "Alveoli" will give a signal. The ventilation can be derived from this signal change. **b**. Upper images: native (unregistered) MR ventilation images during expiration and inspiration. Measurements at the same location are almost impossible while the lung is moving as the same region of interest can not be exactly located. Note the different MR signal and the different size and shape of the lung. Lower images: Registered images. The registration process artificially changes the volume of the lung. An interpolation of the original image intensity values was used to compute the warped image when specific regions are expanded or contracted. The signal changes of the lung are then noted, the size and shape of the thorax stay the same. This way the signal change of each pixel can be measured and ventilation calculated. Note the different MR signal. In contrast to the upper images size and shape of the lung stay unchanged.

As the spatial expansion of the lung and thorax changes with inflation and deflation, it is difficult to define the corresponding lung regions at different times during the respiratory cycle in order to make comparative measurements. We have therefore had to develop an algorithm to adjust the lung margins to a virtually constant shape while preserving the information for signal intensity (Fig. 1).

Calculation of ventilation, represented by a change in air content of a lung region, was carried out according to the following considerations shown as a simplified model in Fig. 1: Briefly, a defined volume (region of interest = ROI) in expiration has a defined MR signal (S_exp_) depending on the content of air and lung parenchyma. At inspiration some of the voxels are replaced by air with a signal equivalent to background noise (S_noise_) resulting in a signal at inspiration S_insp_. Presuming a homogeneous distribution of lung parenchyma within the ROI the total ventilation (V_abs_) in ml air added during inspiration/ml lung parenchyma can now be calculated from the measured signals as

V_abs _= (S_exp_-S_insp_)/(S_exp_-S_noise_)     (1)

Accordingly, the ventilation at any point of the respiratory cycle (V_act_) can be calculated by the actual signal at this point (S_act_) as follows

V_act _= (S_exp_-S_act_)/(S_exp_-S_noise_)     (2)

### Phantom

**Figure 2:**
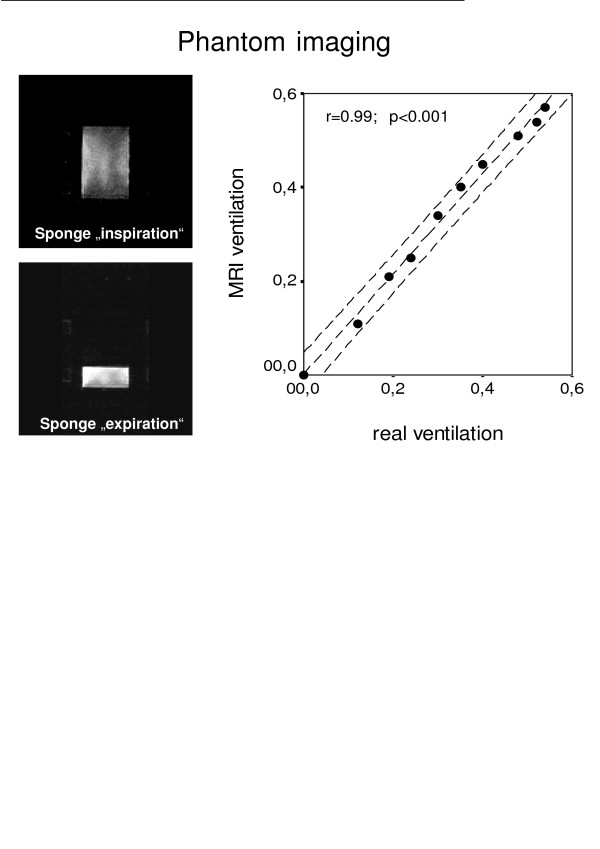
MR image of the phantom at "inspiration" = maximal air content and "expiration" = minimal air content. The graph shows the correlation between the air content in ml air/ml sponge measured by MRI (= MR ventilation) and that calculated from the volume of the sponge (= real ventilation). Dashed lines = regression line and 95% confidence interval.

To simulate lung tissue with its low overall signal intensity and multiple air/tissue interfaces we created a phantom. A cubic sponge of 5 × 12.5 × 20 cm was soaked with silicon oil (Nr.7742, Merck, Darmstadt, Germany) and subsequently squeezed so that its inner surfaces were moistened with a liquid film responsive to MRI. We measured the MR signal serially during slow compression of the sponge (Fig. 2). The "ventilation" expressed as relative air content was measured directly by calculating the change of volume (length as imaged by MRI × width × depth) during compression and compared to the MRI "ventilation" calculated using the equations given above.

### Patients

We performed 99 investigations for 85 patients (60 m/25 f, 3–41 years, mean age 14.1 ± 7.8 y) in a pilot study after approval by the local ethics committee. Written informed consent was given for each investigation by the patients and/or their legal guardians. The patients were divided into five groups. Healthy n = 4, asthma n = 39, cystic fibrosis (CF) n = 19, other lung disease (i.e. pneumonia) n = 17, other disease n = 6.

### Imaging and postprocessing

All MR imaging was performed at 0.2T (Magnetom Open, Siemens, Erlangen, Germany). The fast lung imaging was performed as described previously [7,8]. We chose a true FISP (Fast Imaging with Steady-state Precession) sequence [8-11] modified according to the special needs of functional imaging [1,8]. The initial images of the lung included coronal, lateral and transversal pictures in order to visualize possible morphologic changes and to calculate the lung volume. For "real time" respiration imaging we decreased the spatial resolution to 128 × 128. Repetition time TR 4.2 ms; echo time TE 2.0 ms; flip angle FA 90°; acquisition time TA 1.2 sec/image (including the preparation of the sequence). With this sequence we obtained 50 coronal images over a time period of one minute during slow, maximum amplitude respiration. To overcome the low proton density we increased the slice thickness to 60 mm. The signal was therefore strong enough for clear images of the lung. Conventional pulmonary function tests (PFT) were performed in parallel for 85 investigations. Vital capacity (vc) was chosen for comparison because of the similar breathing technique for vc in PFT and ventilation imaging using MR.

### Registration and measurements

All the images were transferred to a separate workstation. To compensate for breathing motion we used a registration algorithm proposed by C. Chefd'hotel et al. [12,13]. Registration was performed pair wise between all the images and an arbitrary reference image. For each pair [13], this algorithm finds a deformation that maximizes the local cross-correlation between the selected reference and the image being registered. This deformation is modelled as a smooth vector field that gives for each pixel on the reference its corresponding location on the second image. The local cross-correlation criterion was selected for its robustness to intensity changes, signal inhomogeneities, and noise. The algorithm recovers the deformation by composition of small displacements, incrementally maximizing the similarity criterion. This process, which can be seen as the numerical implementation of a transport equation, offers a large capture range, which is required for this application. Given how pathologies can affect breathing motion, we found difficult to use a physical model to constrain the recovered deformation field. Instead, a simple smoothness assumption was used, and proved very effective in practice. This algorithm was selected for its ability to register images without requiring the extraction and selection of anatomical landmarks. Note that during the breathing cycle, parts of the lungs come in and out of the coronal acquisition plane (despite the large slice thickness). This type of deformation can not be captured by the registration method.

**Figure 3:**
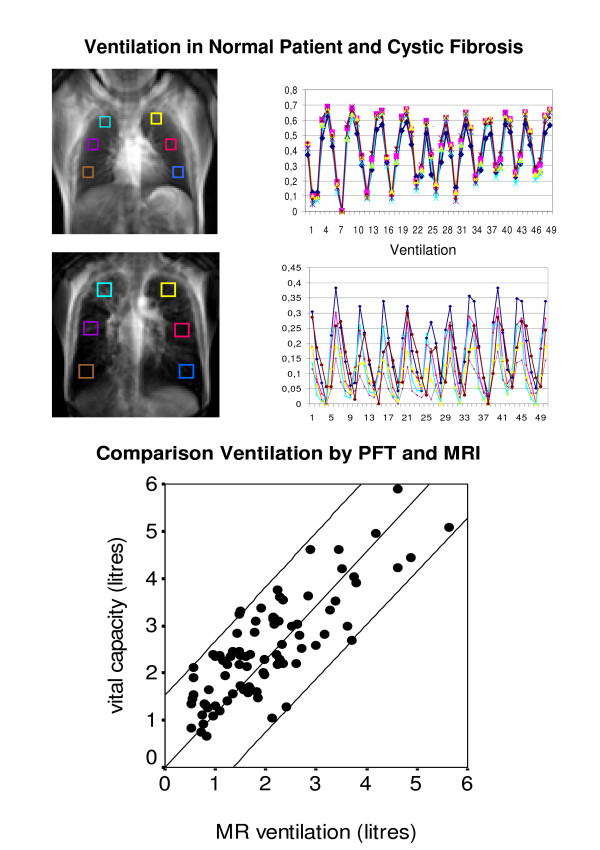
**Upper image and graph**: patient with normal pulmonary function test. On the left, a MR ventilation image is shown with the 6 ROIs (in different colours) used for ventilation measurement, on the right are the ventilation graphs which show the ventilation (= ml air/ml lung parenchyma) in 50 images for each ROI during the respiratory cycles. The 50 measurements span a total of one minute. The colours of the graphs match the colours of the ROIs in the ventilation image to differenciate the 6 lung regions. The ventilation is similar in each of the ROIs. Note the tiring of the young patient (maximal in- and expiration at the beginning only). Ventilation measurements: Right: upper field (turquoise) 0.64 ml/cm^3^; middle field (violet) 0.68 ml/cm^3^; lower field (brown) 0.65 ml/cm^3^. Left: upper field (yellow) 0.67 ml/cm^3^; middle field (pink) 0.68 ml/cm^3^; lower field (blue) 0.62 ml/cm^3^. **Middle image and graph**: patient with cystic fibrosis. The total ventilation is markedly decreased compared to the healthy patient. Additionally, the different lung regions show a very different ventilation, poorest in the left middle field. Ventilation measurements: Right: upper field (turquoise) 0.27 ml/cm^3^; middle field (violet) 0.17 ml/cm^3^; lower field (brown) 0.3 ml/cm^3^. Left: upper field (yellow) 0.2 ml/cm^3^; middle field (pink) 0.32 ml/cm^3^; lower field (blue) 0.38 ml/cm^3^. **Bottom Graph**: Correlation of MR ventilation and vital capacity measured by conventional pulmonary function test (r = 0,8; p ≤ 0,001). Black lines = regression line and 95% confidence interval.

On the registered images we measured signal changes bilaterally in the upper, middle, and lower field for each image throughout the respiratory cycle and calculated the actual ventilation V_act _(equation 2). Total MR ventilation V_abs _was determined by the difference between minimum and maximum air content of the lung over the 50 serial images (equation 1, Fig. 3).

To give a quantitative estimate of total lung ventilation and allow a comparison with PFT results, we performed ventilation measurements in six representative ROIs (Fig. 3). Total lung ventilation was extrapolated by multiplying the mean ventilation (in ml air/ml lung parenchyma) derived from those ROIs with the total lung volume determined by the static thorax MRI scans. The mean ventilation was compared to the conventional PFT of the same day.

### Ventilation maps

To visualize dynamic "real time" ventilation at maximum resolution, we produced ventilation maps, calculating the signal changes between each image over the respiratory cycle. The ventilation was calculated for each pixel and colour-coded red for a decrease in air content (expiration) and green for an increase in air content (inspiration). To assess the ventilation near real-time, a video was produced for each patient showing the increase or decrease of air content in 50 images over 1 minute. Thus the ventilation of the whole lung over the respiratory cycle was evaluated (supplementary videos on request).

## Results

### Phantom imaging

We considered maximum compression of the sponge to be "no ventilation" (0). The lung phantom confirmed that "ventilation" (= the increase of air in the sponge) can be calculated reliably with this MRI based method (r = 0.99; p ≤ 0.001) (Fig. 2).

### Patient imaging

Whole lung ventilation determined by MRI correlated very well with the vital capacity (vc) derived from conventional PFT, with an overall r = 0.8 (p ≤ 0.001) for linear regression (Fig. 3). For healthy subjects r = 1; for asthma r = 0.84; (p ≤ 0.001), for cystic fibrosis (CF) r = 0.8; (p ≤ 0.001), for other lung disease r = 0.74; (p ≤ 0.001), for other disease r = 1 (table 1).

**Table 1:** Comparison of ventilation measurements.

**Diagnosis/Ventilation**	**VC (L) **	**VC (%)**	**FVC (L)**	**FVC (%)**	**FEV1 (L)**	**FEV1 (%)**	**MR ventilation (L)**
**Healthy**	2.33 ± 0.08	92.7 ± 12.7	2.29 ± 0.92	93 ± 13.9	1.98 ± 2.33	96.5 ± 21.8	2.29 ± 0.24
**Asthma**	2.65 ± 1.19	86.9 ± 13.3	2,68 ± 1.21	89.8 ± 11.8	2,39 ± 1.03	97 ± 14.1	2,51 ± 1.45
**CF**	2.51 ± 1.11	66.9 ± 20.3	2.44 ± 1.05	67.1 ± 18.9	1.7 ± 0.85	57.5 ± 25.9	2.86 ± 1.46
**Other lung disease**	2.21 ± 0.79	79.4 ± 16.8	2.2 ± 0.78	80.9 ± 17.5	2.05 ± 0.75	89.8 ± 21.7	2.07 ± 1.09
**Other disease**	1.87 ± 1.58	63.2 ± 6.3	1.87 ± 1.58	67.9 ± 9.4	1.78 ± 1.7	68.3 ± 5.2	2.02 ± 1.59
**All**	2.49 ± 1.08	79.5 ± 18.1	2.49 ± 1.08	81.4 ± 17.9	2.12 ± 0.96	84.3 ± 25.6	2.49 ± 1.38

Conventional pulmonary function measurements in liters and percent of the predicted compared to the ventilation measured by MRI in liters. Mean values ± standard deviation. VC = vital capacity; FVC = forced vital capacity; FEV1 = forced expiratory volume in 1 second.

In addition to the global function parameters corresponding to results using conventional PFT, the six local measurements allowed differentiation of ventilation of the upper, middle, and lower lung regions (table 2). While in healthy persons, the lower fields of both lungs were markedly more ventilated (p ≤ 0.05), this effect was diminished in patients with asthma or CF (not significant; Wilcoxon test for matched pairs) (table 2). This is in agreement with values reported in the literature [14]. The mean ventilation in any of these segments was reduced in asthma patients and was even less in CF patients compared to healthy persons (table 2). For asthma: lower fields p ≤ 0.05; middle and upper fields not significant. For CF: lower fields p ≤ 0.001; middle and upper fields p ≤ 0.05 (Wilcoxon test).

**Table 2:** MRI ventilation measurements.

**Lung region/Diagnosis**	**All patients**	**Healthy**	**Asthma**	**CF**
**Right upper field (ml/ml)**	0.38 ± 0.15	0.52 ± 0.18	0.43 ± 0.14	0.31 ± 0.13
**Right middle field (ml/ml)**	0.39 ± 0.14	0.53 ± 0.16	0.44 ± 0.13	0.32 ± 0.12
**Right lower field (ml/ml)**	0.41 ± 0.14	0.62 ± 0.10	0.45 ± 0.14	0.35 ± 0.12
**Left upper field (ml/ml)**	0.37 ± 0.15	0.50 ± 0.14	0.42 ± 0.15	0.31 ± 0.13
**Left middle field (ml/ml)**	0.38 ± 0.13	0.51 ± 0.18	0.41 ± 0.13	0.33 ± 0.10
**Left lower field (ml/ml)**	0.41 ± 0.14	0.65 ± 0.11	0.43 ± 0.13	0.36 ± 0.11
**Mean (ml/ml)**	0.39 ± 0.13	0.55 ± 0.12	0.43 ± 0.13	0.33 ± 0.11
**Mean FVC (%)**	81 ± 18	93 ± 13	89 ± 12	67 ± 11
**Mean VC (%)**	80 ± 18	93 ± 13	87 ± 13	67 ± 20

Measurement of the local ventilation in 6 ROIs by MRI. The ventilation is given as ml air per ml lung parenchyma. Note the differences of local ventilation for different groups of patients. For comparison FVC and VC of the conventional pulmonary function test. FVC% = forced vital capacity in % of the expected. VC (%) = vital capacity in % of the expected.

### Ventilation maps

**Figure 4:**
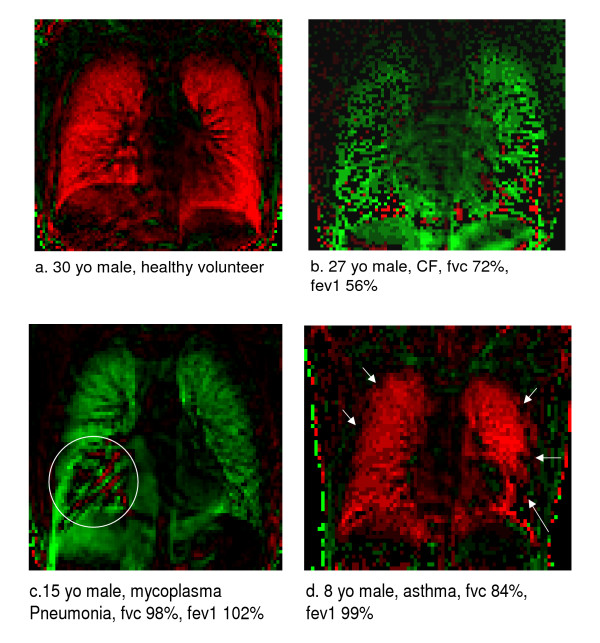
Ventilation map images. One image selected from the ventilation map video. Increase in air content is coded red (inspiration); decrease is coded green (expiration). In image b and c note the artifacts in the region of the diaphragm which occur secondary to the registration process which registers the lungs only. However, the lung is still clearly outlined. **a**. healthy person in inspiration. Note the homogeneous contribution of air in inspiration. **b**. patient with cystic fibrosis. Note the multiple ventilation defects (black areas) and the poor overall ventilation (patchy ventilation pattern and little colour coded areas). **c**. patient with mycoplasma pneumonia. Despite normal global lung function a large ventilation defect (Circled black area) is noted in the right lower field where conventional imaging shows a pneumonic infiltrate. **d**. patient with asthma and good fev1 in the pulmonary function test. Despite good fev1 this patient may require more efficient treatment as there is still an impairment of local ventilation peripherally and in the left lower field (arrows).

In healthy persons, ventilation maps showed a homogeneous distribution while impaired ventilation was seen mostly in the lower lung regions and peripherally in patients suffering from asthma, less frequently in the upper or middle fields. In CF patients, diffusely distributed ventilation deficits were frequently observed (Fig. 4). In most cases, these typical patterns allowed differentiation of the disease categories simply by viewing the ventilation maps.

In our pilot study 25 investigations were performed (healthy n = 1; asthma n = 16; CF n = 3; other lung disease n = 5) in persons who had a normal global PFT (vc and fev1 > 90%). However, only 5 (20%) of these were rated to have no ventilation defects in the ventilation mapping. 5 (20%) had ≤ 3, 13 (52%) > 3, and 2 (8%) diffuse ventilation defects. On the other hand, of 15 investigations (CF n = 12; other lung disease n = 2; other disease n = 1) of patients with pathologic PFT (vc and fev1 < 70%), 3 (20%) had > 3 and 12 (80%) diffuse ventilation defects.

## Discussion and conclusion

These data indicate that the method presented here could be used to visualize local destruction of the lung long before an impairment can be detected by conventional PFT. Native ventilation lung MRI appears therefore to be just as good as other methods, such as helium-3 imaging, with which similar results have been reported [15-18]. Our method may even be superior with respect to cost-effectiveness and feasibility in clinical routine. One examination takes only 10–15 minutes. The method could be established for any low-field MR-scanner, no other special equipment or patient preparation is needed. Investigations can be repeated without exposing the patient to radiation or contrast agents, making it a perfect tool for studying diseases which require patients to have regular scans, such as asthma. Another significant advantage is the rapid collection of images. For ventilation mapping, images are taken about one a second, so several breathing cycles can be imaged within 1 min. This is important for studying diseases like asthma, in which local ventilation may change rapidly due to airway closure, as well as for the examination of less cooperative patients such as young children. In addition, investigations of physiological and pathophysiological processes and conditions can be performed without the application of a contrast agent which might itself alter the normal function of the lung. The advantages of local ventilation imaging have been previously discussed [19-23]. The novelty of this method is the *quantitative *measurement of the ventilation which can even be calculated for each voxel.

One of the basic assumptions of the method presented in the paper is that the MR signal of the lung parenchyma changes during ventilation mostly due to the different air content. Significant changes in T2* due to influences like perfusion and others would therefore diminish the accuracy of the ventilation calculation. From previous studies of our group and others it is well known, that T2* is relatively long (about 19 ms) at 0.2 T [1] and the signal decay is flat during the short time of measurement. Therefore the assumption of a relatively robust (against small changes in T2*) signal measurement was made, knowing this possible source of error which made a phantom and clinical validation of the method necessary.

According to our data the ventilation values seem reliable as they correlate closely with the lung function study which is the "gold standard" of ventilation assessment. However, as shown in figure 3, at high vital capacities MR ventilation may slightly underestimate the ventilation measured by global lung function tests. This problem may be caused by the slice thickness which in patients with a high vc should in future studies probably be adjusted to the thorax diameter to image as much of the lung as possible. Up to now the algorithm for morphing the lung works by matching the boundaries only. Therefore regions that expand different in the slice direction may be under- or overestimated. However the ventilation measured in the different fields is not dependent on the posterior-anterior (pa) expansion as it is measured in ml air per ml parenchyma. Therefore regions that expand more will have a higher ventilation regionally as seen in the lower lung fields. The overall ventilation however may be affected as it was calculated using the mean thorax diameter. In a future step more precise measurements of the different thorax expansions in the pa direction could overcome this problem.

At this early stage of the study, the image transformation was, in part, done manually. For registration and ventilation mapping, one image was selected on which to perform the further computational steps. At this point a critical observation of the results by the investigator is necessary to check the plausibility of the registration process. In future, automated image selection and processing should be performed easily. By imaging multiple slices, 3D ventilation maps could also be achieved in the near future. In conclusion, the method presented here has the potential to become a major improvement of pulmonary assessment for clinical purposes as well as for research.

## Competing interests

Authors Rupprecht and Zapke are owners of a pending pct patent application and interested in contacting potential licensees.

The remaining authors declare that they have no competing financial interest.

## Authors' contributions and acknowledgements

We thank Prof. Dr. W. Rascher for his support, L. Fiedler and M. Jakobi for the patient investigations, L. Naehrlich and T. Zimmermann for patient recruitment.

T. Rupprecht was the initiator of the study and wrote the ventilation map tool. He had great part in data interpretation and preparation of the manuscript. M. Zapke, and HG. Topf were responsible for patient recruitment, performed the patient investigations, the data acquisition, postprocessing and interpretation as well as the preparation of the manuscript. R. Kuth, M. Deimling and P. Kreisler had part in the sequence modelling and hard- and software acquisition. M. Rauh had part in the phantom design. C. Chefd'hotel and B. Geiger programmed the registration tool.

## Supplementary Material

Additional File 1 **Supplementary video 1**. Native MR video of a 15 year-old patient with asthma. 50 images during slow respiration. Note the signal changes of the lung parenchyma. Click here for file

Additional File 2 **Supplementary video 2**. Ventilation video of the same patient. Inspiration is coded green, expiration red. Note the poor ventilation in the left lower field. Pulmonary function test in this patient was normal. Click here for file
